# popRange: a highly flexible spatially and temporally explicit Wright-Fisher simulator

**DOI:** 10.1186/s13029-015-0036-4

**Published:** 2015-04-11

**Authors:** Kimberly F McManus

**Affiliations:** Departments of Biology and Biomedical Informatics, Stanford University, Stanford, CA 94305 USA; Departments of Biomedical Informatics, Stanford University, Stanford, CA 94305 USA

**Keywords:** Population genetics, Python, R, Genetic simulators

## Abstract

**Background:**

Sequencing and genotyping technology advancements have led to massive, growing repositories of spatially explicit genetic data and increasing quantities of temporal data (i.e., ancient DNA). These data will allow more complex and fine-scale inferences about population history than ever before; however, new methods are needed to test complex hypotheses.

**Results:**

This article presents popRange, a forward genetic simulator, which incorporates large-scale genetic data with stochastic spatially and temporally explicit demographic and selective models. Features such as spatially and temporally variable selection coefficients and demography are incorporated in a highly flexible manner. popRange is implemented as an R package and presented with an example simulation exploring a selected allele’s trajectory in multiple subpopulations.

**Conclusions:**

popRange allows researchers to evaluate and test complex scenarios by simulating large-scale data with complicated demographic and selective features. popRange is available for download at http://cran.r-project.org/web/packages/popRange/index.html.

**Electronic supplementary material:**

The online version of this article (doi:10.1186/s13029-015-0036-4) contains supplementary material, which is available to authorized users.

## Background

Recent advances in sequencing and genotyping technology have led to dramatic reduction in cost and increased accuracy of DNA sequencing. This advance has led to the creation of large repositories of spatially explicit genetic data and increasing quantities of temporal data (i.e., ancient DNA). Furthermore, data continue to be generated at an unprecedented rate; 10X more sequences are generated every year [1-3].

Simulators are ubiquitous in population genetics and current simulators tend to focus on either large-scale data or demographic and environmental stochasticity (Table 1).

**Table 1:** **Comparison of population genetic simulators**

**Feature**	**SPLATCHE2** [16]	**SimAdapt** [6]	**quantiNEMO** [7]	**SFS_CODE** [4]	**SLiM** [5]	**popRange**
**Simulation method**	Coalescent	Forward-Time	Forward-Time	Forward-Time	Forward-Time	Forward-Time
**Data type**	SNPs, STRs, DNA sequences, RFLPs	SNPs, STRs	SNPs, STRs	SNPs, DNA sequences	SNPs, DNA sequences	SNPs
**Interface**	Command-line, GUI	Command-line, GUI, accessed via R package RNetlogo	GUI	Command-line	Command-line	R package
**Many SNPs (>100)**	No	No	Allowed, but very slow [6]	Yes	Yes	Yes
**Population structure**	Friction, migration rates (one rate per population)	Dispersal distance (one rate per population)	Migration rates, stochastic founding/extinction of populations	Migration rates, speciation, domestication & admixture events	Migration rates	Population grid based, migration rates, stochastic founding/extinction of populations
**Population dynamics**	Logistic growth	Logistic growth	Logistic growth	Logistic & exp. growth, step size changes	Step size changes	Logistic growth, Allee effect, step size changes
**Natural selection**	No	Fixed values	Many models, spatially & temporally varying	Fixed values, gamma, normal & 3-point mass models	Fixed values, gamma & exponential distributions	Fixed values, gamma distribution, spatially & temporally varying
**Linkage**	Yes	No	Yes	Yes	Yes	No

This table presents a brief comparison of population genetic simulators. For more in-depth comparisons, see [8,9,17].

Software such as sfs_code [4] and SLiM [5] allow simulation of large segments of DNA and integrate a wide range of parameters, such as recombination, migration and selection. These simulators allow extraction of haplotypes at various time points to explore time-series genetic trends. However, they require specification of divergence times, founder population sizes and deterministically set migration rates, which limit their ability to model stochastic demographic events. For example, modeling range expansions are difficult to simulate, as populations cannot stochastically populate the world.

Other simulators allow populations to form and diverge in a more stochastic manner than those described above. However these simulators focus on a small number of independently segregating loci. One of the most flexible simulators, SimAdapt [6], allows, among many features, temporally variable gene flow barriers, differences in fitness between populations, and different carrying capacities. Another simulator, quantiNemo [7], allows the simulation of spatially and temporally explicit selection coefficients, but requires the user to set starting allele frequencies and runs very slowly on even mid-sized data [8]. Simulators in this category are typically unable to generate the large quantity of single nucleotide polymorphisms (SNPs) and still lack flexibility with respect to spatially and temporally variable parameters.

A main use of a new generation of simulators is to allow researchers to evaluate and test hypotheses generated from the data, with flexible scenarios [9]. Most modern population genetic analyses, including principal component analysis (PCA), large-scale inference of demography (i.e. ∂*a*∂*i* [10]) and ancestry analyses (i.e. ADMIXTURE [11]) require the generation of a large number of independently segregating SNPs.

popRange bridges this gap by simulating complex demographic scenarios with large-scale genetic data. These simulators are necessary to interpret current genetic data in more realistic demographic scenarios. Though popRange does not simulate linkage, independently segregating loci are sufficient for many large-scale analyses.

This software provides a simulation framework for modeling highly probabilistic spatial and temporal population dynamics. To date, no existing simulator incorporates both stochastic spatially and temporally explicit scenarios and chromosome-scale data. This grid-based population structure model allows spatial and migration flexibility, such as in simulations of arbitrary landscape barriers. However, information from both types of data in simulations is essential to gain insight to realistic dynamic processes on the genome.

## Implementation

### Technical details

popRange is implemented as an R package and requires R [12], Python 2.7.× or Python 3.2.×-3.4.× [13], the Python package NumPy [14], and the R package findpython [15]. As an R package, it can run on any operating system.

### Simulation overview

popRange is a highly probabilistic Wright-Fisher forward population genetic simulator. Specifically, it incorporates 1) large scale data (many SNPs, populations, and individuals), 2) a grid-based population structure, 3) a wide variety of spatially and temporally explicit stochastic demographic parameters, and 4) a variety of output file formats.

Simulations are based on a user defined population grid, starting population sizes, and starting SNP model. Alternatively, users can use the output of a previous simulation to set up the initial populations. Thus multiple runs can be set so most parameters can be temporally variable, as well as spatially variable. Each generation goes through a set of phases (in this order):

1. *Extinction*: Each generation, each population can become extinct with probability set by the user.
2. *Migration:* Migration rates are spatially and temporally explicit, allowing the simulation of a wide range of landscapes. Note that migration is highly probabilistic; rates are the probability each individual migrates in each generation. The number of migrants from each population is determined by a binomial distribution and migration probability. These probabilities may allow a random adjacent destination population to be chosen or they may be specific with respect to initial and final populations.
3. *Mutation:* Mutations are based on the infinitely many sites model. The number of mutations introduced into each population in each generation is drawn from a Poisson distribution parameterized by:
  $$ \lambda =\mu *g*N $$
  where *μ* is the mutation rate parameter, *g* is the number of base pairs in the genome, and *N* is the population size.
4. *Selection:* When a mutation is introduced, a selection coefficient may be placed on the new allele. Selection coefficients may be fixed values or may be drawn from a gamma distribution.
5. *Population growth:* Populations may grow logistically or may experience instantaneous population size changes. For logistic growth, the growth rate, *r*, is drawn from a normal distribution with a mean and variance provided by the user. This *r* is used in the logistic growth equation:
  $$ {N}_t=r*{N}_{t-1}*\frac{1-{N}_{t-1}}{K}*\frac{N_{t-1}-A}{K}, $$
  where *N* is the population size, *K* is the carry capacity and *A* is the Allee effect.
6. *Drift/Reproduction:* Populations may be haploid or diploid and random mating is assumed within each hermaphroditic population.
7. *Output results:* When all generations are complete, output may be written to a variety of file formats, including Geneland, PLINK, and GENEPOP (Additional file 1: Section 5).

## Results and discussion

This framework combines simulators commonly used in ecology, which incorporate stochastic demographic scenarios, such as population growth and contraction, and stochastic founding and extinction of populations, with simulators more commonly used in population genetics, which include many SNPs. It also incorporates more advanced features such as spatially and temporally explicit selection.

### Runtime

Runtime scales linearly with the number of base pairs and the number of individuals (see Additional file 1). For reference, simulating a 100 kb sequence in a 4 × 4 population grid (16 populations) with 100 diploids per population, 0.01 migration rate between adjacent populations, 1.1*E*-8 mutation rate per generation and 1000 generations completes in a bit under 4 minutes.

### Accuracy

This software was evaluated for accuracy through comparisons with theoretical expectations of heterozygosity, fixation probabilities of new mutations, and Fst (Additional file 1: Section 6).

### Example simulation

**Figure 1:**
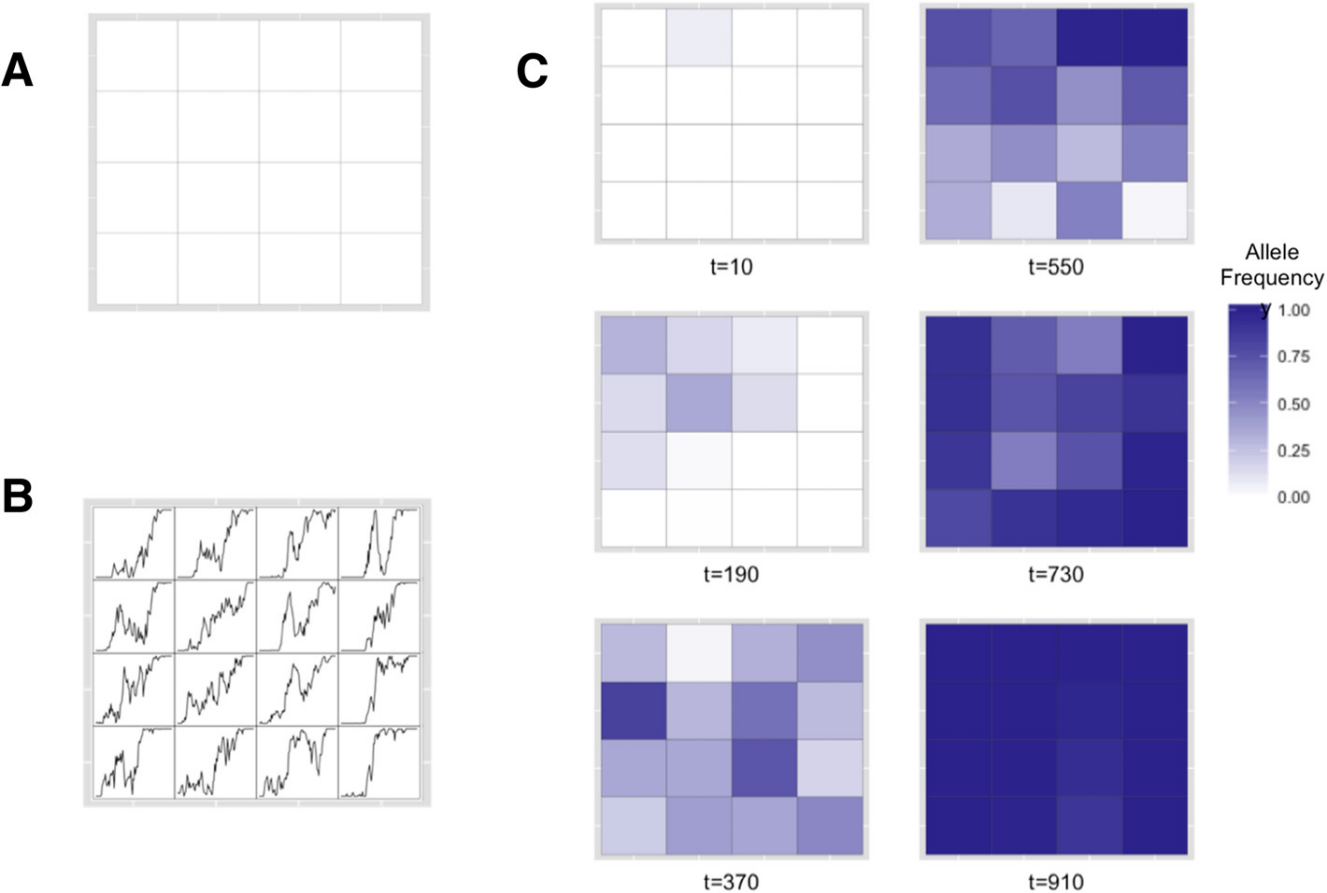
**Allele frequencies and trajectories over time in an example simulation. A)** Grid of simulated populations. **B)** Trajectory of an allele that originated in population (1,2) in generation 8. Limits of each y-axis are 0 (new allele not present) and 1 (new allele fixed). The x-axis limits are 0 to 1000 generations. **C)** Heat maps of the allele frequency in each population at six time points.

Figure 1 shows an example simulation. In this simulation, a 4x4 grid of populations was simulated. Each population started with 100 diploid individuals. The migration rate is set to 0.01, meaning that on average one individual migrates from each population during each generation. Individuals can migrate to any adjacent population. The mutation rate was set to 1.1*E* − 8 per site per generation and the genome size is 5,000,000 base pairs. This figure follows the evolution of a neutral allele that originated in population (1,2) at generation 8 and eventually fixes in all populations in generation 950. It is interesting to note that the population it originates in is not the population in which the frequency initially rises. In (4,4) the allele is introduced and has a characteristic fixation trajectory; it is initially at low frequency, but rapidly fixes after an initial frequency increase. However, in (1,4) the allele almost reaches fixation before a dramatic decrease and recovery. It would be interesting to explore these dynamics in greater depth and understand the role they may play in real populations. Furthermore, popRange allows simulation of complex features, such as temporally and spatially varying selection and landscape barriers. Results from comparisons of models incorporating these features can inform expectations of patterns in real data and add to our understanding of evolutionary dynamics.

## Conclusion

popRange allows users to simulate spatially and temporally explicit scenarios with chromosome-scale data efficiently for the first time. Features such as spatially and temporally variable selection coefficients are incorporated in a flexible manner. This software allows for large-scale analyses and comparisons of these complex, stochastic models and is implemented in R, facilitating ease-of-use. I expect that this software will fill a gap and help researchers better make use of the increasing geographically explicit genomic data that is being accumulated for diverse group of organisms.

## Availability and requirements

**Project name:** popRange

**Project homepage:**http://cran.r-project.org/web/packages/popRange/index.html

**Direct Download link:**http://cran.r-project.org/src/contrib/popRange_1.1.2.tar.gz

**Operating systems:** Linux, Mac OS X, Windows

**Programming languages:** R, Python 2.7.× or Python 3.2.×-3.4.×

**Other requirements:** NumPy (python package), findPython (R package)

**License:** MIT

**Any restrictions to use by non-academic users:** no licenses required.

## Additional file

Additional file 1:
**Supplementary Manual.** Supplementary manual for the program that provides in-depth details on running the program.
