# Supplementary material for: popRange: a highly flexible spatially and temporally explicit Wright-Fisher simulator
Source: Source Code Biol Med. 2015 Apr 11;10:6. doi: 10.1186/s13029-015-0036-4 (PMC4399400; doi:10.1186/s13029-015-0036-4)
Supplement: Additional file 1: — Supplementary Manual. Supplementary manual for the program that provides in-depth details on running the program. [file 13029_2015_36_MOESM1_ESM.pdf]

Table of contents

1. INTRODUCTION **2**
1.1 WHAT POPRANGE CAN DO 2
1.2 HOW POPRANGE WORKS 2
2. GETTING STARTED **4**
2.1 DOWNLOAD AND INSTALLATION 4
3. INPUT **5**
3.1 REQUIRED PARAMETERS 5
3.2 TEMPORALLY VARYING PARAMETERS 6
3.3 SELECTION PARAMETERS 7
3.4 GROWTH PARAMETERS 7
3.5 OTHER PARAMETERS 8
3.6 OUTPUT PARAMETERS 9
4. EXAMPLES **10**
4.1 EXAMPLE 1 10
4.2 EXAMPLE 2 10
4.3 EXAMPLE 3 11
4.3 EXTRA EXAMPLES 11
5. OUTPUT **12**
5.1 RESULTS FILE 12
5.2 GENELAND FILE 12
5.3 GENEPOP FILE 13
5.4 PLINK FILE 14
5.5 RECORDTRAG FILE 14
6. PROGRAM VALIDATION **15**
6.1 HETEROZYGOSITY 15
6.2 FIXATION PROBABILITIES 16
6.3 FST 17
7. RUNTIME MEASUREMENTS **18**
7.1 SCALING BY NUMBER OF POPULATIONS 18
7.2 SCALING BY NUMBER OF BASE PAIRS 19
7.3 SCALING BY NUMBER OF INDIVIDUALS PER POPULATION 20
8. GENERAL ISSUES **20**
9. ACKNOWLEDGEMENTS **20**
10. REFERENCES **21**

1. Introduction

1.1 What popRange can do

This package is a forward-in-time Wright Fisher population genetic simulator. It is highly flexible and probabilistic, incorporating stochastic spatially and temporally explicit scenarios and chromosome scale data. It allows the simulation of large numbers of individuals, SNPs, and populations. This combines simulators commonly used in ecology, which incorporate stochastic demographic scenarios, such as stochastic founding and extinction of populations, with simulators more commonly used in population genetics that incorporate large numbers of SNPs.

1.2 How popRange works

This is a grid-based forward-in-time Wright-Fisher population genetic simulator. It is highly flexible and incorporates many parameters, as described below. This spatially explicit simulator is based on a user-defined grid of populations (Supplementary Figure 1). Most parameters can also be temporally variable. popRange can read its own output file to start a new simulation where the previous one left off. By running the simulation multiple times, a user can cause temporal changes to many of the parameters.

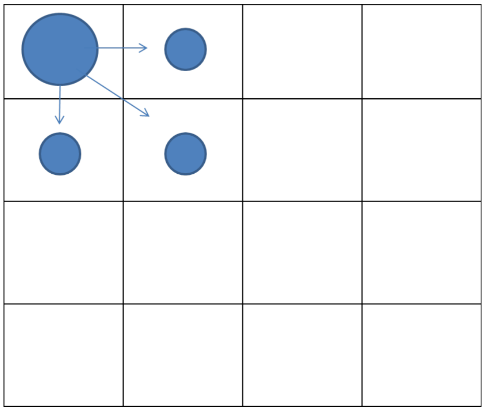

Supplementary Figure 1: Each grid point represents a population habitat. In this example, the initial population (top left) sends small groups of migrants to the adjacent habitats. Over a long period of time, this process results in the dynamic peopling of the grid.

Steps of the simulation

Setup
This step initializes the world.

Each generation (in this order):

Catastrophe

Each generation, each population becomes extinct with probability equal to the catastrophe parameter set by the user. If a population becomes extinct, its population size immediately goes to 0.

Migration

Migration is grid-based. All populations can have the same migration rate or each set of populations can have its own migration rate.

If the migration rate parameter is a single value, the code first determines the number of individuals migrating away from each population (pulled from a binomial distribution with probability equal to the migration rate parameter). Then each migrant is randomly assigned a random adjacent population to migrant. The population grid has closed boundaries. A non-edge population has eight adjacent population grid points.

If migration rate parameter is a matrix (migration rates are specified for particular pairs of populations), the number of migrants from the initial to final population is chosen from a binomial distribution with the probability given. These initial and final populations are not required to be adjacent to each other and may be located anywhere on the grid.

NOTE: The migration rate parameter is the probability of an INDIVIDUAL migrating. Thus if it is haploid, it's the probability of each haploid migration and if individuals are diploid, it's the probability of a two chromosomes migrating. In all cases, the order of population migrations is randomly shuffled to ensure no false patterns emerge.

Mutation

Mutations are based on the infinitely many sites model. The number of mutations introduced into each population in each generation is drawn from a Poisson distribution parameterized by:

$$\lambda = \mu * g * N$$

where μ is the mutation rate parameter, g is the number of base pairs in the genome, and N is the population size.

Selection

In diploid individuals, the fitness of the new allele is equal to $1 - s$ in homozygotes and $1 - hs$ in heterozygotes. In haploid individuals, the fitness of the new allele is $1 - s$.

When a mutation is introduced, a selection coefficient, s, and a dominance coefficient, h, may be placed on the new allele. The user may define a fixed selection coefficient s or a gamma distribution (with parameters *alpha* and *beta*) for s to be drawn from. Like most parameters in this framework, selection parameters are very flexible, and is also possible to select spatially and temporally varying values. The default s is 0, indicating the mutation is neutral, and the default h is 0.5, indicating that heterozygotes (ex. Aa) have a fitness in between the two homozygotes (ex. AA and aa).

Drift
Population growth
Populations can maintain a constant size, experience instantaneous population size changes, or experience logistic growth. If logistic growth parameters are set, the new population size is determined at this point. The growth rate, *r*, is drawn from a normal distribution with the mean and variance provided by the user. This *r* is used in the logistic growth equation:

$$N_t = r * N_{t-1} * \frac{1 - N_{t-1}}{K} * \frac{N_{t-1} - A}{K}$$

where *N* is the population size, *K* is the carry capacity and *A* is the Allee effect.

Reproduction
First, if any population has less than 2 individuals at this point, the population goes extinct. Allele frequencies in the remaining, new populations are then calculated. The new frequencies of neutral SNPs are drawn from binomial distributions with probability equal to current SNP frequency. Frequencies of selected SNPs are adjusted to their expected frequencies in the next generation. The new SNP frequencies are then drawn from these adjusted frequencies.

Lose Zeroes
If SNP_Model = 1, and the derived allele is no longer present in any population, the allele is removed from the simulations. This is a technical mechanism to reduce simulation time.

Output results
When all generations are complete, output is written to files chosen by the user.

2. Getting Started

2.1 Download and Installation

The R package is available for download from CRAN at http://cran.r-project.org/web/packages/popRange/index.html. This contains an R package that calls Python scripts. It requires R, Python, and the Python package NumPy to run.

Step 1: Download Python
(Windows users only, should already be installed on Macs)
This software was built with Python 2.7.6, but multiple versions of Python 2.7 and Python 3.2-3.4 should be compatible. Mac computers come preinstalled with Python. Windows users may need to download Python, which is available at https://www.python.org/downloads/windows/. Download either Python 2.7.* or Python 3.4.x. (It should also be compatible with Python 3.2-3.3.)

Step 2: Download NumPy
NumPy is a popular Python package for scientific computing. It is available here: http://www.numpy.org/.

Step 3: Download R
R available for download here: http://www.r-project.org/. We also recommend using the GUI RStudio, available here: http://www.rstudio.com/products/rstudio/

Step 4: Download popRange
popRange can be downloaded from the R website via this link: http://cran.r-project.org/web/packages/popRange/index.html. Alternatively, the following command can be entered into R:

install.packages('popRange')

This package currently has just one main command, popRangeSim, which is the command to run the simulations. It depends on the package "findPython", which should be downloaded automatically with the install.packages command.

Step 5: Try it out!
Everything should be set up now. Try out some of the examples and walkthrough this manual for more information. Questions, comments and suggestions can be directed to Kimberly McManus at kfm@stanford.edu.

3. Input

3.1 Required Parameters

Below includes all required parameters.

NOTE: There are two main possible SNP models.

- *Model 0*: User provides starting SNP frequencies. For this model, the user must provide the total number of SNPs (nSNPs) and the starting SNP frequencies (SNPs_starting_freq).
- *Model 1*: Number of SNPs and starting SNP frequencies are determined via a standard neutral model. If this model is chosen, the user must provide a mutation rate (mut rate) and genome size (gSize).

**Parameter**	**Data Type**	**Description**	**Example**
world	matrix	This matrix grid defines the grid of populations. Each point in the matrix defines a potential population.	world = matrix(0,nrow=3,ncol=3)<br>world[1:2,1:2]=1<br>world[3,2:3] = -1

		A 1 indicates a population may exist at that point. -1 indicates no population can exist at that grid point. This allows the user to define a population grid that is not rectangular.	world=<br>1 1 1<br>1 1 1<br>1 -1 -1
nGens	int	Number of generations for the simulations to run.	nGens = 100
popSize	Matrix or int	Starting population size. If int, every existing population has the same size. If matrix, each population size is defined separately.<br><br>Input must be an integer >= 0 or a matrix of integers >= 0.	Ne = matrix(0, nrow=3, ncol=3)<br>Ne[1,] = c(30,50,10)<br>Ne[2,1:2] = c(10,5)<br><br>Ne =<br>30 50 10<br>10 5 0<br>0 0 0
SNP_Model	int	There are two options. The first option (0) indicates that the user will provide starting SNP frequencies. The second option (1) determines starting SNP frequencies according to the standard neutral model with user provided genome size and mutation rate.	SNP_Model = 1
gSize	int	Number of base pairs in the simulated sequence.<br><br>(Required if SNP_Model = 1)	gSize = 90000
mutRate	float	Mutation rate per generation per site.<br><br>If generating sequence data, 1.2*10**-8 mutations/bp/generation is a possible setting.<br><br>(Required if SNP_Model = 1)	mutRate = 1.2*10**-8
nSNPs	int	Number of SNPs, if SNP_Model = 0.<br><br>(Required if SNP_Model = 0)	nSNPs = 200
SNPs_starting_freq	Int or matrix	The initial frequency of the SNPs. If int, all SNPs are given the same starting frequencies. If matrix, columns 1 and 2 indicate the first and last SNPs. Column 3 indicates the allele frequency of these SNPs.<br><br>(Required if SNP_Model = 0)	SNPs_starting_freq = matrix(0, nrows=3, ncols=3)<br>SNPs_starting_freq = c(1,100,0.3)<br>SNPs_starting_freq = c(101,200,0.2)<br><br>SNPs_starting_freq=<br>1 100 0.3<br>101 200 0.2

3.2 Temporally varying Parameters

Most parameters can be made temporally variable by outputting the first simulation run. Then changing the relevant parameters, reading in the file from the first simulation run, and running a second simulation. This can be done as many times as necessary (see Section 4.3 for an example).

3.3 Selection Parameters

There are two possible selection models. The first draws selection coefficients from a gamma distribution. In this implementation, every SNP has a selection coefficient that is drawn from the gamma distribution. In the second selection model, the user defines specific selection coefficients for each SNP. Each SNP can have the same selection coefficient, or the user may provide a matrix with selection coefficients. NOTE: Provide EITHER an s input OR gamma_shape, gamma_scale values.

Furthermore, the user may implement spatially varying selection coefficients with the sDiff option.

**Selection Parameters**	**Data Type**	**Description**	**Example**
gamma_shape, gamma_scale	Floats	Shape (aka alpha, k) and scale (aka theta) parameters to the gamma distribution.<br><br>In human European populations these have been estimated at shape=0.206, scale=1/15400 (Boyko et al. 2008).	gamma_shape = 0.17<br>gamma_scale = 8
s	Float or matrix	If numeric, all SNPs have the same selection coefficient. If matrix, first 2 columns define starting and ending SNP. The 3rd column is the selection coefficient for those SNPs.<br><br>DEFAULT: 0	s = matrix(c(1,101,100,200, 0.01,0.02), nrow=2, ncol=3)<br><br>s=<br>1 100 0.01<br>101 200 0.02
h	Float	Dominance parameter. (Only relevant for diploids).<br><br>DEFAULT: 0.5	h = 0.6
sDiff	Matrix	Defines the selection coefficients for each SNP in each population. This allows for spatially variable selection coefficients. The first line of the matrix consists of the strings 'sSNP' and 'fSNP'. These are the first and last SNPs with a specific set of selection coefficients. The coordinates of each population are subsequently listed.	sDiff= matrix(0,nrow=4, ncol=6)<br>sDiff[1,] = c('sSNP', 'fSNP', '00', '01', '10', '11')<br>sDiff[2,] = c(1,100,0.01,0.02,0.03,0.04)<br>sDiff[3,] = c(101,200,0.04,0.03,0.02,0.01)<br><br>sDiff =<br>sSNP fSNP 00 01 10 11<br>1 100 0.01 0.02 0.03 0.04<br>101 200 0.04 0.03 0.02 0.01

3.4 Growth Parameters

popRange implements logistic growth and instantaneous population size changes.

Instantaneous size changes can also be simulated by running one simulation at the first size. You can use this output file as input for the next simulation with a different population size.

Ex. The below simulations has 100 diploids for 10 generations, and then instantaneously shrinks to 50 diploids for the next 10 generations.

First run:
popRangeSim(world=1, popSize=100, nGens=10, nSNPs=500, SNPs_starting_freq=0.5, SNP_model=0,outfile="ExampleRun")

Second run (This uses the output file from the first run and changes the size. Be sure to add "results.gen#" to the input file though!).:
popRangeSim(world=1, popSize=50, nGens=10, infile="ExampleRun.results.gen10", outfile="ExampleRun2")

**Growth Parameters**	**Data Type**	**Description**	**Example**
K	Int or matrix	Carry capacity. If int, all populations have same carry capacity. If matrix, each population has its own carrying capacity.<br><br>DEFAULT: 100	K = 1000
A	Int or matrix	Allee Effect. If int, all populations have same Allee effect. If matrix, each population has its own Allee effect. Set to 0 if you don't want to incorporate the Allee effect.<br><br>DEFAULT: 0, no Allee effect	A = 10
rMean	Float or matrix	Mean r (from exponential growth equation). Float between c(0,1). If int, all populations have the same r. If matrix, each population has its own r.<br><br>DEFAULT: 0, no growth	rMean = 0.4
rVar	Float or matrix	Variance in r. Float between c(0,1). If int, all populations have the same variance. If matrix, each population has its own r. Set to 0 if you want r to be exactly rMean each generation.<br><br>DEFAULT: 0	rVar = 0.4

3.5 Other Parameters

**Other Parameters**	**Data Type**	**Description**	**Example**
diploid	binary	Ploidy of individuals. Two options: haploid and diploid. If TRUE, diploid. If FALSE, haploid.	Diploid = TRUE

		DEFAULT: TRUE	
migration	Float or matrix	Probability of each individual migrating each generation. If float, every population has same migration rate. Each migration randomly picks an adjacent viable grid point to migrate to (diagonals included).<br><br>Alternatively, a matrix can be provided. The first 2 columns are the coordinate of the initial population. The next two columns are the coordinates of the final population. The fifth column is the migration rate. Population pairs not included are assumed to have no migration.<br><br>NOTE: Think of the x coordinate as the horizontal coordinate and the y as the vertical coordinate.<br><br>DEFAULT: 0	migration = matrix(c(0,1,0,0,0,0,1,0,0.01,0.01), nrow=2, ncol=5)<br><br>Pop1x pop1y pop2x pop2y p<br>0 0 0 1 0.01<br>1 0 0 0 0.02
catProb	Float or matrix	Probability of population extinction each generation. If a float, all populations have the same catProb. If matrix, each population has its own catProb.<br><br>DEFAULT: 0	catProb = 0.01
inFile	string	If you have already ran a simulation, you can use the results file as input. This allows for temporally variable parameters and to output the state of the population at various time points.	infile = 'results.1'

3.6 Output parameters

A results file is always output. This can be read back into the program.

**Output parameters**	**Data Type**	**Description**	**Example**
outfile	string	A string of the outfile names.	outfile = "first_run"
GENEPOP	Binary	If TRUE, write results to GENEPOP formatted file	GENELAND = FALSE
GENELAND	Binary	If TRUE, write results to GENELAND formatted file	GENEPOP = FALSE
PLINK	Binary	If TRUE, write results to an PLINK formatted file	PLINK = FALSE
recordTrag	Int	If n, this records the trajectory of all of the	recordTrag =10

		alleles n generations and outputs them to a single file.<br><br>NOTE: If you have many SNPs or populations, this option may significantly increase simulation time. Also if you are using SNP_model=1, alleles that have a frequency of 0 in all population will be dropped from simulation and thus will stop being recorded in this file. This decreases simulation time.<br><br>DEFAULT = 0, only outputs results at end	#This would record the state of every allele every 10 generations

4. Examples

4.1 Example 1

In this scenario, we are simulating a 3x3 grid of populations for 1000 generations. Each population starts with 200 diploid individuals. Each individual has a 0.01 probability of migrating away from their populations. There are 1000 SNPs that all have a starting frequency of 0.5, and the program outputs the standard "results" file (see Section 5.1), as well as a PLINK file (see Section 5.2).

Command:

```
mat = matrix(1,nrow=3,ncol=3)
popRangeSim (world = mat, popSize = 200, diploid = TRUE, nGens = 1000, mig = 0.01, SNP_model = 0, nSNPs = 1000, SNPs_starting_freq = 0.5,  outfile= "test_outfile", PLINK=TRUE)
```

4.2 Example 2

In this example, we simulate a grid of 15 populations (no population can exist at (1,1)) with spatially varying selection coefficients. We are also recording the state of all alleles every 10 generations (recordTrag = 10). The migration rate is set to 0.01, so each individual has 0.01 probability of migrating away from their population in each generation.

Commands:

```
mat = matrix(1,nrow=4,ncol=4)
mat[1,1] = -1 #This makes a population grid where a population exists at every point, except [1,1]
sDiff= matrix(0,nrow=3, ncol=6) #Since there is no population at [1,1] we set (0,0) to  no selection.
sDiff[1,] = c('sSNP', 'fSNP', '00', '01', '10', '11') #'sSNP' is the starting SNP and 'fSNP' is the ending SNP
sDiff[2,] = c(1,100,0,0.02,0.03,0.04)
sDiff[3,] = c(101,200,0,0.03,0.02,0.01)

popRangeSim (world = mat, popSize = 100, sDiff = sDiff, nGens = 100, mig = 0.01 , SNP_model = 0, nSNPs = 200, SNPs_starting_freq = 0.1, h = 0.5, outfile = "test_outfile", recordTrag = 10, diploid = TRUE)
```

4.3 Example 3

This is a more complicated example.

Here we simulate 2 populations, where there is 0.01 probability of each individual migrating 1 → 2 each generation and a 0.02 probability of each individual migration from 2 → 1 each generation. Then, after 500 generations, this reverses.

NOTE: The output file adds ".results.gen__" to the file name you select. Be sure to add this to the file name when you read in the file in the second simulation.

```
mat = matrix(1,nrow=2,ncol=1)
mig_mat = matrix(c(0,1,0,0,1,0,0,0,0.01,0.02), nrow=2, ncol=5)


#Pop1x pop1y pop2x pop2y p
#0     0     0     1     0.01
#0     1     0     0     0.02


popRangeSim (world = mat, popSize = 100, s = 0.01, nGens = 50, mig = mig_mat , SNP_model = 0, nSNPs = 100,
SNPs_starting_freq = 0.1, h = 0.5, outfile = "test_outfile", diploid = TRUE, GENELAND = TRUE)

mig_mat = matrix(c(0,1,0,0,1,0,0,0,0.02,0.01), nrow=2, ncol=5)
popRangeSim (world = mat, popSize = 100, s = 0.01, nGens = 50, mig = mig_mat, SNP_model = 0, nSNPs = 100,
SNPs_starting_freq = 0.1, h = 0.5, outfile = "test_outfile", diploid = TRUE, infile = "test_outfile.results.gen50")
```

4.3 Extra Examples

Here we simulation a 3x3 population grid (9 populations), which have varying starting population sizes. There is a 0.01 probability of each individual leaving their population each generation (for an adjacent population). This model is initialized with a standard neutral model (SNP_model=1) with a 100,000 bp sequence, 1*10^-4 mutation rate per site per generation. Each population has a carrying capacity of 50 and the Allee effect parameter is set to 2. The average growth rate, rMean is 0.4 with a variance of 0.4.

```
##Ex 4.
## Testing popSize, SNP Model 1, K, A, rMean, rVar, catProb
## A 3x3 grid of populations

mat = matrix(1,nrow=3,ncol=3)
Ne = matrix(0, nrow=3, ncol=3)
Ne[1,] = c(30,50,10)
Ne[2,1:2] = c(10,5)
popRangeSim(world=mat, popSize=Ne, nGens=50, mig=0.01, SNP_model=1,
        gSize=100000, mutRate=1*10^-4, K=50, A=2, rMean=0.4, rVar=0.4,
        catProb=0, outfile="test_outfile")
```

Below we output the state of every allele every 10 generations (recordTrag=10)

```
#Testing recordTrag
mat = matrix(1,nrow=3,ncol=3)
popRangeSim (world = mat, popSize = 50, diploid = TRUE, nGens = 100,
        mig = 0.01, SNP_model = 0, nSNPs = 1000, SNPs_starting_freq = 0.5,
```

```
outfile= "test_outfile_6", PLINK=TRUE, recordTrag=10)
```

Below we set a gamma distribution for the distribution of fitness effects. Note that when there are many selected alleles, the simulations may slow down significantly. We initially with a standard neutral model (SNP_Model=1) where all 9 populations have a starting size of 50.

```
##Testing gamma distribution of selective factors
mat = matrix(1,nrow=3,ncol=3)

popRangeSim(world=mat, popSize=50, nGens=100, mig=0.01, SNP_model=1,
        gSize=100000, mutRate=1*10^-8, gamma_shape=0.17, gamma_scale=1, outfile="test_outfile7")
```

5. Output

There are multiple possible output formats. File structures are explained below.

5.1 Results file

The results file is a CSV file:
Line 1: max # pops in x direction, max # pops in y direction, # SNPs
Line 2: haploid population size of 1st pop, x coordinate of 1st pop, y coordinate of 1st pop.
→ If population is diploid, this number should be divided by 2 to get the # diploids.
Line 3: SNP positions. (Just a 0 based list from 0 to nSNPs-1)
Line 4: Selection coefficients for each SNP in that population
Line 5: # of each allele in the population
Line 6: The generation where the allele arose. (If set from the beginning, this will be 0)

EXAMPLE:

```
1, 2, 5             #Two pops.  5 SNPs
3, 0, 0             #3 individuals, coordinates of this pop: (0,0)
0,  1, 2, 3, 4              #SNP positions
0.0, 0.0, 0.0, 0.0, 0.0    #No selection
3, 3, 2, 1, 0               #Allele frequencies
0, 0, 0, 0, 0               #All alleles were set at the beginning of this particular simulation
5, 0, 1                     #5 individuals, coordinates of this pop: (0,1)
0,  1, 2, 3, 4              #SNP positions
0.0, 0.0, 0.0, 0.0, 0.0    #No selection
0, 2, 4, 4, 1               #allele frequencies
0, 0, 0, 0, 0       #All alleles were beginning of this simulation and thus arose at gen 0
```

5.2 GENELAND file

The GENELAND file has the two alleles. There is one line per individual. If individuals are diploid, there is a / separating the two alleles. “01” is the ancestral allele and “02” is the derived allele.

EXAMPLE:

01/01 02/02 01/02 01/01 01/01
02/02 02/01 01/02 01/01 01/01
02/01 02/02 02/02 01/02 02/01

5.3 GENEPOP file

First line: Arbitrary
Second line: List of all loci IDs
Third line: The word “POP”
Next, there is a line for each individual in the first population. Each line starts with an individual ID defined by: POP[x cor of pop][y cor of pop]_[individual number]
The list of alleles has “01” is derived and “02” is ancestral.

EXAMPLE:

Arbitrary first line
Loc1, Loc2, Loc3, Loc4, Loc5
POP
POP00_1, 0101 0202 0102 0101 0101
POP00_2, 0202 0201 0102 0101 0101
POP00_3, 0201 0202 0202 0102 0201
POP
POP01_1, 0202 0101 0102 0101 0202
POP01_2, 0202 0101 0102 0101 0202
POP01_3, 0202 0101 0102 0101 0202
POP01_4, 0202 0101 0102 0101 0202
POP01_5, 0202 0101 0102 0101 0202

GENEPOP also comes with a coordinate file. There is one line per individual, and it contains coordinates of the population to which each individual belongs.

EXAMPLE:

00
00
00
01
01

01
01
01

5.4 PLINK file

See the PLINK website for more detailed information (http://pngu.mgh.harvard.edu/~purcell/plink/data.shtml#ped)

The PLINK output format contains two files: a .PED file and a .MAP file. The .PED has one line per individual. If diploid, both alleles are present on the same line. The first six columns are: FamilyID, IndividualID, PaternalID, MaternalID, Sex, Phenotype. Most of these columns are not relevant to these simulations, so they are set to 0 or -9.

EXAMPLE:

POP00 0 0 0 -9 -9 1 1 1 2 1
POP00 1 0 0 -9 -9 1 1 2 1 1
POP00 2 0 0 -9 -9 2 1 2 1 1
POP01 0 0 0 -9 -9 2 1 1 1 1
POP01 1 0 0 -9 -9 1 2 1 1 1
POP01 2 0 0 -9 -9 2 1 1 1 1
POP01 3 0 0 -9 -9 2 1 1 1 1
POP01 4 0 0 -9 -9 1 1 1 1 1

5.5 RecordTrag file

Currently, there is one file that contains the whole history for every allele.
Columns: (1) Generation, (2)siteNumber (siteNum), (3)generation allele arose, (4) selection coefficient of allele, (5) # derived copies in first population (0,0), (6) # derived copies in second population (0,1) (7…) Another column for each subsequent population in the simulations.

gen	siteNum	genAlleleArose	s	pop00	pop00ne	pop01	pop01ne	pop02	pop02ne
0	0	0	0	16	30	30	50	0	0
0	1	0	0	18	30	30	50	0	0
0	2	0	0	12	30	21	50	0	0
0	3	0	0	17	30	26	50	0	0
0	4	0	0	16	30	29	50	0	0
0	0	0	0	16	30	30	50	0	0
0	1	0	0	18	30	30	50	0	0
0	2	0	0	12	30	21	50	0	0
0	3	0	0	17	30	26	50	0	0

0	4	0	0	16	30	29	50	0	0

6. Program Validation

To ensure accuracy of these simulations, multiple metrics have been compared to their theoretical expectations.

6.1 Heterozygosity

Observed and expected heterozygosity were compared over 1000 generations. (Expected heterozygosity based on Hartl & Clark 2007):

$$H_t = (1 - \frac{1}{2N+1})^t * H_0$$

where H is the heterozygosity, N is the population size, t is the number of generations, and H_0 is initial heterozygosity. Below simulations were based on one population of 100 diploid individuals. There are 500 SNPs with starting SNP frequencies of 0.5. Fifty simulations were run to determine mean and confidence intervals.

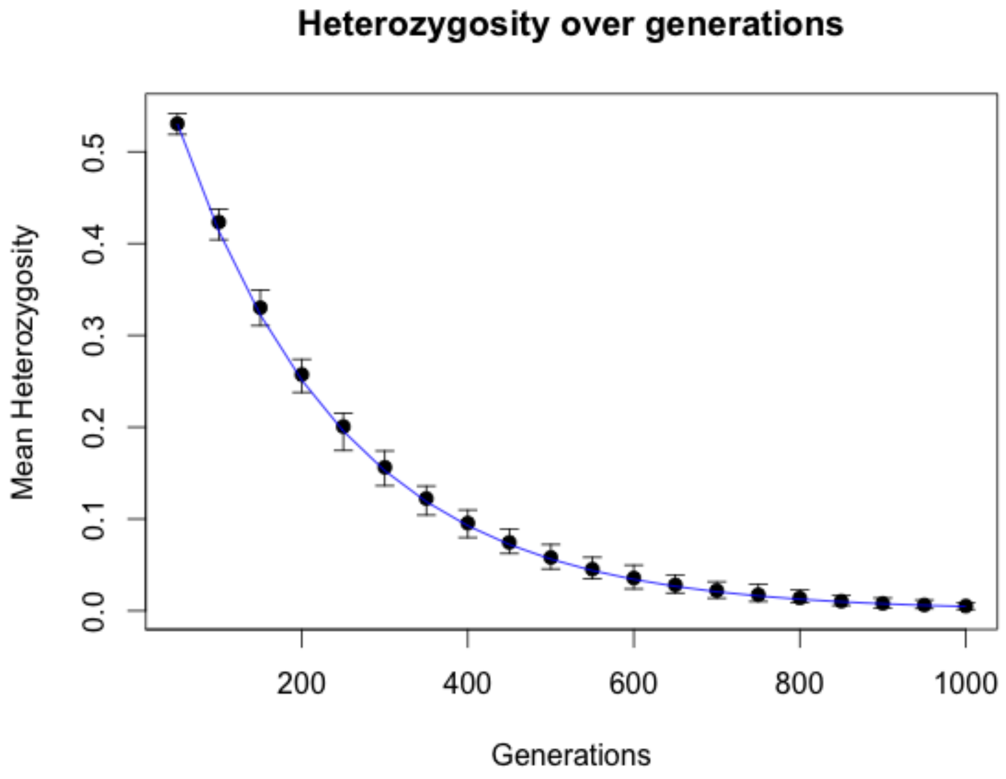

Supplementary Figure 2 Expected (blue) vs. observed (black) heterozygosity over 1000 generations. Results are based on one population of 100 diploid individuals with 500 SNPs all starting at a frequency of 0.5. Mean and 95% confidence intervals determined via 50 independent simulations.

6.2 Fixation Probabilities

The probability of fixation of a new mutation, based on its selection coefficient and population size, has been determined by Kimura (Kimura, 1962):

$$P(s, N) = \frac{1 - e^{-s}}{1 - e^{-2Ns}}$$

where N is the effective population size and s is the selection coefficient.

This equation assumes randomly mating diploid population with constant population size. It also assumes each mutation evolves independently and new mutations have a heterozygous fitness of 1 + 0.5s.

Below results show fixation probabilities in the simulations closely align to their theoretical expectations. Each simulation was run with one population of 100 diploid individuals and each SNP had a starting frequency of $1/2N$. For each selection coefficient, simulations were run until 50 SNPs had reached fixation. The standard deviations are determined from Poisson counts.

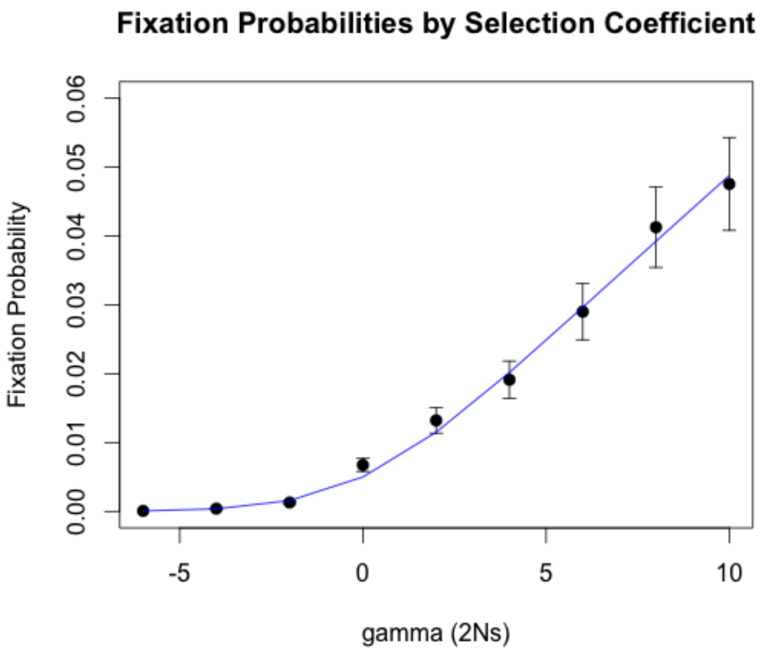

Supplementary Figure 3. Expected fixation probability (blue) and observed fixation probability (black).

6.3 Fst

Expected Fst over generations is determined by (Hartl and Clark, 2007):

$$F(t) = \frac{1}{2N} * (1-m)^2 + \left(1 - \frac{1}{2N}\right) * (1-m)^2 * F(t-1)$$

where N is the population size and m is the migration rate. This equation assumes random mating within the populations.

Two different simulation models were explored. The first model has nine populations with no migration. All populations have 50 diploid individuals and 500 SNPs with starting SNP frequency of 0.5

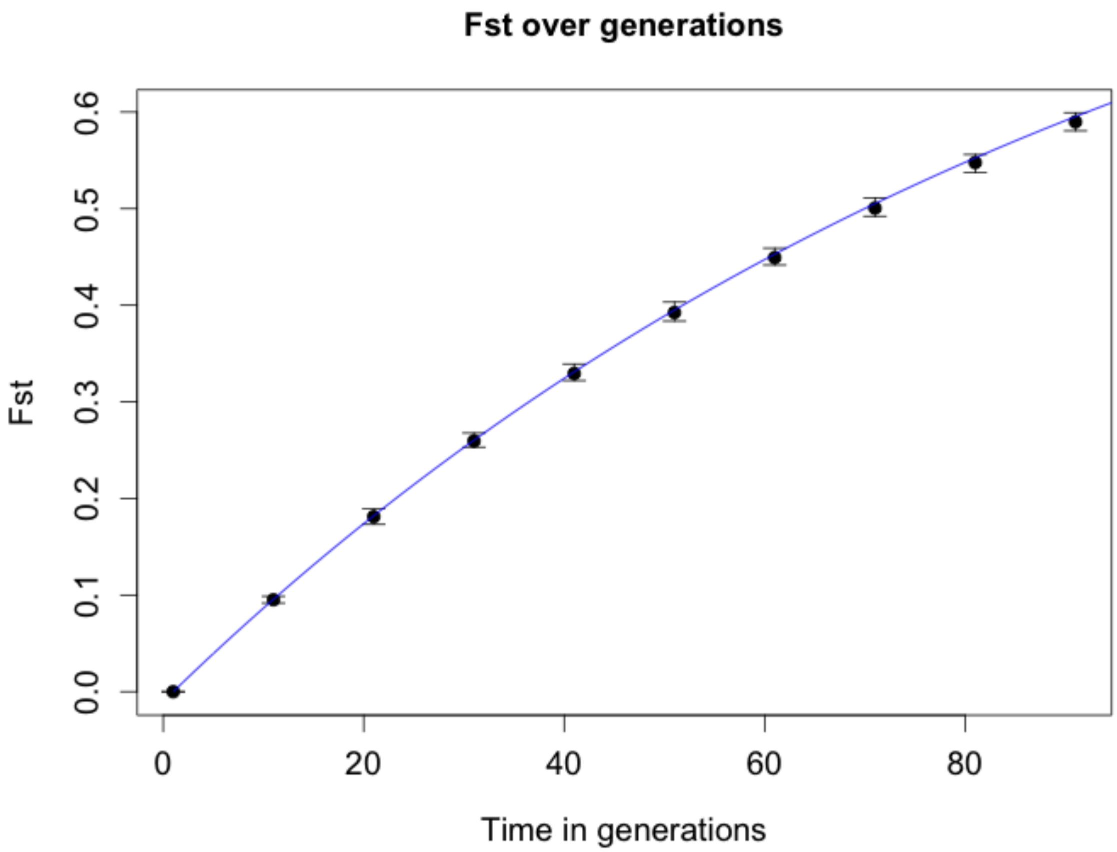

Supplementary Figure 4 Expected Fst (blue) and observed Fst (black) over 100 generations. Observed Fst means and confidence intervals determined via 50 independent simulations of four populations.

The second model has nine populations with 0.001 migration rate. All populations have 50 diploid individuals and 500 SNPs with starting SNP frequency of 0.5. Mean and confidence intervals were determined from 50 simulations.

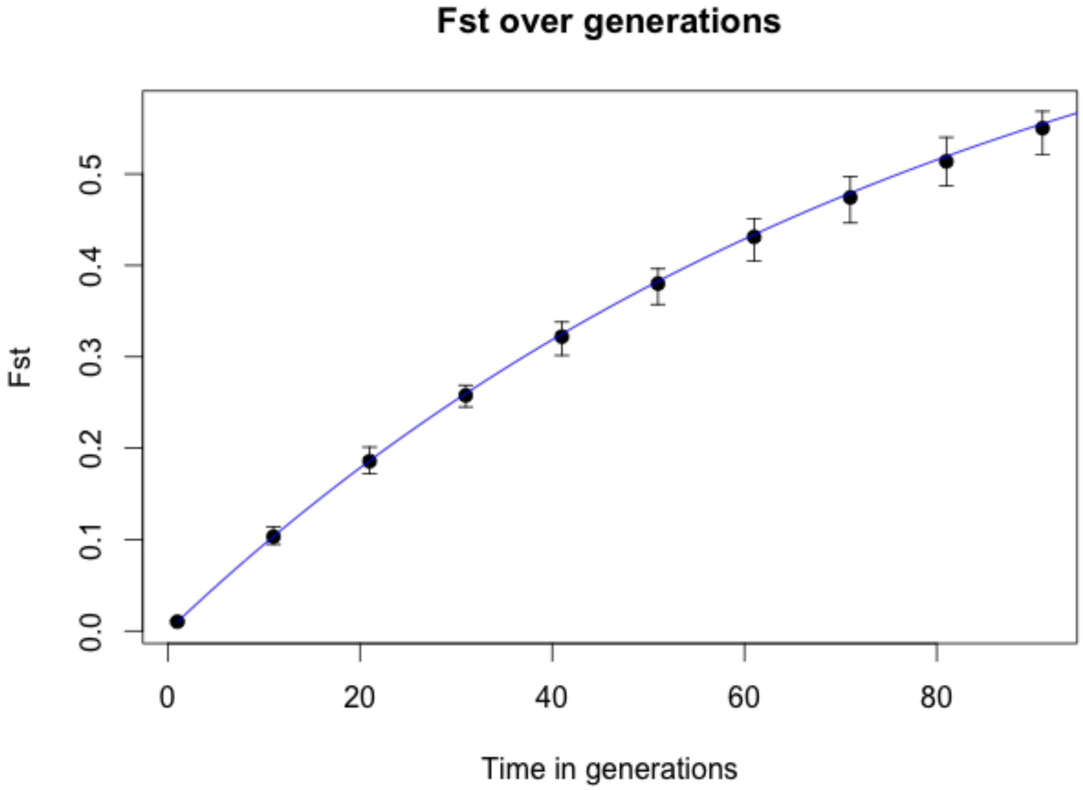

Supplementary Figure 5 Illustrates theoretical (blue) and observed (black) Fst with 50 diploid individuals and 0.001 migration rate. Observed results determined via simulations of nine populations. Mean and confidence intervals determined through 50 independent simulations.

7. Runtime Measurements

All runtime measurements were conducted on a 2012 MacBook Pro with 8 GB of memory and a 2.9 GHz Intel Core i7 processor.

7.1 Scaling by number of populations

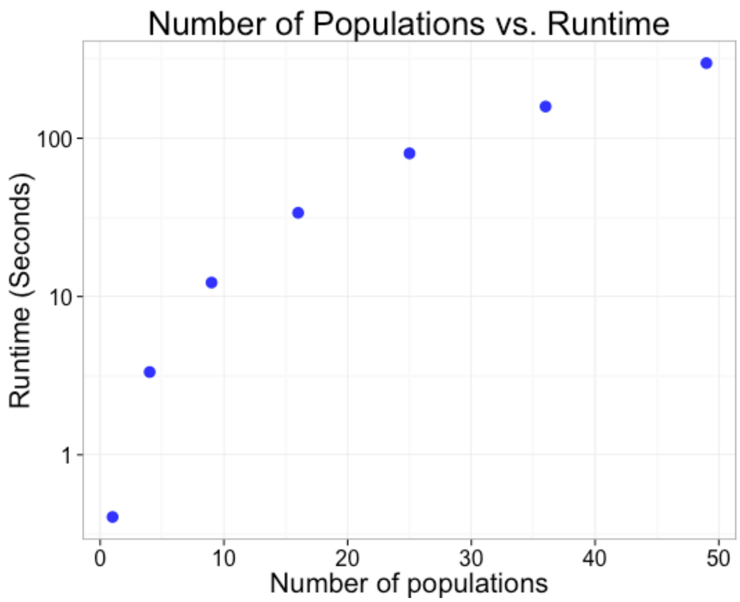

Supplementary Figure 6: Runtime measurements based on the number of populations.

Simulation parameters
Number of generations = 1000, size of each population = 100 diploids, standard neutral model, μ=1.1*e*-8, sequence length=0.1 MB, migration rate = 0.01 (on average, 1 individual leaves each population each generation)

Population grid sizes = 1x1, 2x2, 3x3, 4x4, 5x5, 6x6, 7x7

7.2 Scaling by number of base pairs

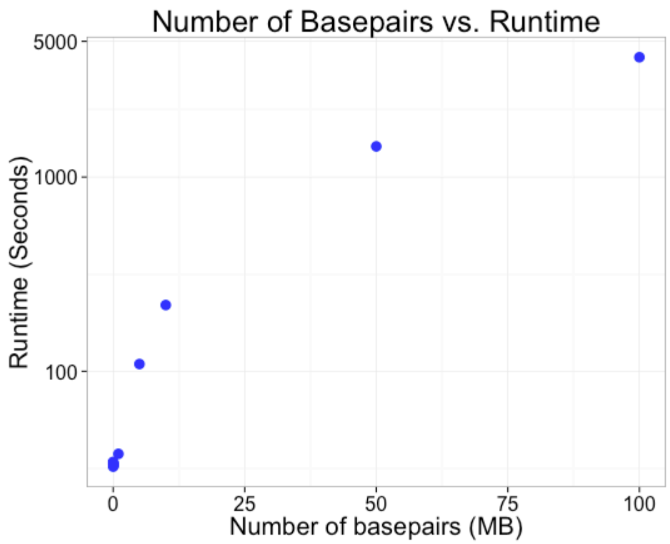

Supplementary Figure 7: Runtime measurements based on the number of base pairs.

Simulation parameters

Number of generations = 1000, number of populations = 16 (4x4 grid), size of each population = 100 diploids, standard neutral model, μ=1.1*e*-8, migration rate = 0.01 (on average, 1 individual leaves each population each generation)

7.3 Scaling by number of individuals per population

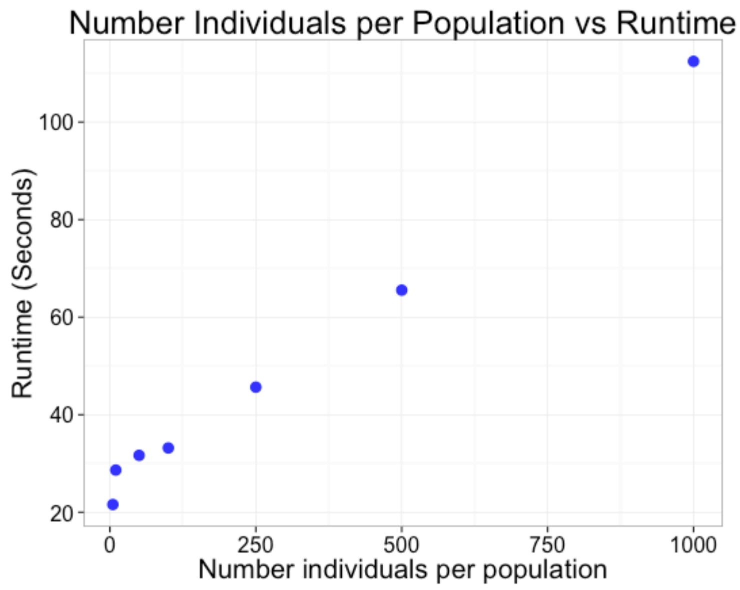

Supplementary Figure 8: Runtime measurements based on the number of individuals in each population.

Simulation parameters
Number of generations = 1000, number of populations = 16 (4x4 grid), standard neutral model, μ=1.1*e*-8, sequence length = 0.1 MB, migration rate = 0.01 (on average, 1 individual leaves each population each generation)

8. General Issues

As this software allows simulation of very complex scenarios, it is important to ensure that your parameters are consistent with each other. The input population matrix overrides some inconsistencies. If, for example, a migration rate is set for a population grid point that cannot exist according to the population matrix, then the migration rate will be ignored. Other inconsistencies may result in an error. However, it is very important to check that the parameters are consistent.

9. Acknowledgements

The author would like to thank Dr. Carlos Bustamante and Dr. Omar Cornejo for valuable feedback on the simulation framework and manuscript. This work was funded by the NIH training grant #5T32GM007276-38 and a Stanford Center for Computational, Evolutionary and Human Genomics (CEHG) fellowship.

10. References

Boyko et al. (2008). Assessing the evolutionary impact of amino acid mutations in the human genome. *PLOS Genetics.* DOI: 10.1371/journal.pgen.1000083.

Hartl DL, Clark AG. (2007) Principle of Population Genetics, Fourth Edition. Sinauer Associates, Inc.

Kimura M. (1962). On the probability of fixation of mutant genes in a populations. *Genetics.* 47:713-719.
